# Why all clinical guideline recommendations are ‘Conditional’

**DOI:** 10.1002/gin2.12013

**Published:** 2024-04-05

**Authors:** Peter C. Wyer, John Gabbay, Edward H. Suh

**Affiliations:** ^1^ Department of Emergency Medicine Columbia University New York New York USA; ^2^ Faculty of Medicine University of Southampton Southampton UK

**Keywords:** health equity, mindlines, practice guidelines

## Abstract

- The impact of clinical guidelines on practitioner behaviour and clinical care remains in doubt.
- The acceptability of a 5‐ or 10‐year delay in producing a ‘strong’ clinically important recommendation is unlikely to hold.
- Practitioners think holistically, in contrast to the analytical thinking that characterizes clinical research.
- Rapidly accessible, flexible, ‘knowledge‐in‐practice‐in‐context’ cannot be simply replaced by a focused piece of new research or guideline.

## 1 INTRODUCTION

Important advances in clinical guideline development have emerged in the three decades since David Eddy first introduced the term ‘evidence‐based’ into the medical literature.^1^ In the early 2000s, the Grading of Recommendations Assessment, Development and Evaluation (GRADE) initiative introduced rating of evidence quality using a range of considerations, rather than mere study‐design criteria.^2^ In 2011, a US Institute of Medicine report defined standards for the trustworthiness of clinical guidelines, which required a systematic review of the literature and called for the incorporation of representative stakeholders into guideline panels.^3^ A parallel report called for routine consideration of evidence from observational studies and randomized trials,^4^ already a provision of the GRADE system. These advances gained traction,^5^ and over the course of the last 10 years, they raised expectations regarding guideline quality.

Yet, the impact of clinical guidelines on practitioner behaviour and clinical care remains in doubt. A 2003 landmark study^6^ found that only around half of eligible patients in the United States were receiving guideline‐recommended care. Surveys continue to demonstrate non‐adherence and practitioner reluctance to follow guidelines.^7^, ^8^ Recently, the experience of the coronavirus disease (COVID) pandemic exposed additional barriers and impediments to the adoption and adherence to guideline recommendations in day‐to‐day practice. Understanding these barriers in the context of the social processes surrounding healthcare delivery and decision‐making could be key to increasing the real‐world impact of the clinical guideline enterprise. This is the thesis of this commentary.

## 2 THE COVID REVELATIONS

COVID‐19 exposed rifts, inequities and weaknesses in many aspects of society, including flaws in the linkage between research, guidelines and clinical practice. Foremost was the difference between the timescale of change in clinical practice and that of the production, synthesis and incorporation into guideline recommendations of clinical research. This was particularly evident during the first months of the pandemic when the severity of a still unfamiliar illness was at its height.^9^

An historical mission of the Evidence‐Based Medicine movement was to close the research‐to‐practice gap. In 1993, a widely cited article demonstrated a 10‐year gap between the emergence of evidence supporting a specific intervention and its acceptance as standard care.^10^ The table illustrates the current existence of a similar time lag between the initial planning of a trial capable of definitively changing the evidence base underpinning a guideline recommendation and the incorporation of its findings into a revision (Table 1). Over this time, clinical practice may well change in ways that threaten the practical applicability of the recommendation.^9^

**TABLE 1:** Illustrating the process through which a potentially definitive clinical trial leads to the incorporation of a strong recommendation into published clinical guidelines.

Phase of production	Time allowance^a^
Trial planning and funding decision	1–2 years
Conduct of funded trial	3–5 years
Incorporation of trial results into systematic reviews	2 years
Incorporation of synthesized evidence into published guideline recommendations	2 years
Total time	8–11 years

^a^ These time estimates are derived from information contained in funding applications for US federal research grants, posted production intervals of systematic reviews in the Cochrane Library and in published guideline reports.

The pandemic demonstrated that current technology has transformed the possible speed of research production, synthesis and dissemination.^11^ Given the prospect of such an acceleration, the acceptability of a 5‐ or 10‐year delay in producing a ‘strong’ clinically important recommendation is unlikely to hold.

In fact, strong recommendations have become a rarity within published guidelines. Inevitably, evidence ratings based on diverse criteria, such as those used in the GRADE system, tend to be lower than when based only on the relative strength of research designs. Correspondingly, the assigned strength of recommendations also usually decreases. This phenomenon is demonstrable. For example, across successive editions of existing guidelines before and after the adoption of GRADE, the rating of evidence and recommendations frequently decreases even though the evidence is unchanged.^2^ Furthermore, strong recommendations that do emerge are likely to be discordant, that is, supported by a low level of evidence. This potentially disinclines end users to follow published guidelines and has also caused dismay within the guideline literature.^12^, ^13^ The tension between the lack of definitive research evidence and practitioners' need for guidance comes to the fore in times of healthcare crisis, as was seen during the recent pandemic.^9^, ^14^

The pandemic exposed two other impediments within the research‐to‐guideline‐to‐practice trajectory. The first relates to disparities in healthcare delivery and outcomes across racial, ethnic and socioeconomic divides. Not only studies of COVID outcomes but also other research show the inherent complexity of the underlying factors.^15^ For example, attempts to remedy the disparity in treatment of patients with chronic kidney disease might actually increase the risk of poor clinical outcomes in certain populations.^16^ Threats to health equity risk undermining public trust in healthcare and have been acknowledged in the guideline literature.^17^ The ‘Evidence‐to‐Decision (EtD)’ framework, an initiative within the GRADE consortium,^18^ is a noteworthy response to increased concerns regarding equity and complexity within the GRADE consortium. It offers a structured approach to helping guideline panels anticipate potential disparities in the impact of recommendations, overcome challenges to adoption or identify the need for adaptation. EtD is particularly applicable to the context of recommendations involving complex interventions that depend on circumstances and health‐related behaviours that vary between different racial, ethnic and socioeconomic groups and communities. However, the framework is elaborate; it is more likely to be employed within population‐based contexts than to individual patient‐level decision‐making.^19^

A second obstacle to smoothly linking research to practice through guideline development, also highlighted by the COVID experience, is that even well‐designed studies of health interventions frequently focus on specific, narrow inquiries rather than on whether an intervention is beneficial within the social context in which it is delivered. This question of wider context is explored in the following segment.

## 3 THE SOCIAL DETERMINANTS OF KNOWLEDGE USAGE

COVID‐19 exacerbated longstanding challenges to the conventional model of clinical guideline development, dissemination and adoption. To help meet those challenges, guideline producers need to consider what is known about the actual process through which practitioners use their products. Over two decades ago, Gabbay and le May's ethnographic studies of highly rated clinical practitioners illuminated how information from research and clinical guidelines is processed through the social interactions of clinical care.^20^, ^21^ These findings were consistent with studies of how clinicians process and use information in day‐to‐day practice.^22^ They highlight the fact that practitioners think holistically, in contrast to the analytical thinking that characterizes clinical research. Clinical researchers are trained to focus on single, well‐defined options and to limit the role of competing or confounding factors through, for example, single hypotheses, limited patient eligibility and institutionally specific protocols. Similarly, guideline developers seeking an evidence‐based approach frequently echo researchers' orientation and may distance their efforts from the changeable, contextual complexities of real‐world decision‐making. In contrast, the variable context of clinical reasoning and decision‐making requires a complex interplay of considerations stemming from a wide range of domains, including patient‐specific factors, potential unplanned effects, multimorbidities, social circumstances, the availability of resources such as tests and treatments, and economic or organizational demands and constraints.

Ethnographies of practitioners suggest that effective clinicians cope with this variable complexity by developing what Gabbay and le May called ‘clinical mindlines’. Mindlines can be characterized as socially situated, contextualized and shared cognitive structures analogous to, but much broader than, the notion of ‘illness scripts’ well known to educational psychologists.^21^ Mindlines are ‘the internalized, collectively reinforced and often tacit guidelines that are informed by clinicians’ training, by their own and each other's experience, by their interactions with their role sets, by their reading, by the way they have learnt to handle the conflicting demands, by their understanding of local circumstances and systems and by a host of other sources’.^21^ Developed throughout a practitioner's clinical career, mindlines constitute rapidly accessible, flexible, ‘knowledge‐in‐practice‐in‐context’ that cannot be simply replaced by a focused piece of new research evidence or guideline. Rather, such new information is subjected to the processes of collective and individual mindline development.^20^, ^21^ These processes transform the distillations of research evidence and guidelines into a carefully blended internalized cocktail of practical, contextualized knowledge whose individual evidential ingredients, drawn from many sources, can no longer be easily distinguished but which stand a much better chance of rapidly hitting the spot for any particular individual patient.

These ethnographic findings have been extended to encompass the use of research information not only in the context of health policy making and education^23^, ^24^ but also of guideline production. Observations of well‐regarded guidelines‐development panels in three countries have highlighted how they inevitably utilize their own clinical mindlines as they process the research evidence.^25^ A guideline panel may, depending on its members and their methods of working,^26^ formulate variable blends of the distilled evidence.

The value of guidelines depends on the context of their use. The mindlines concept explains how and why clinicians ultimately transform the research‐based information within clinical guidelines into knowledge that is practically useful in diverse circumstances. Much has been done to try to improve the contextual relevance of research evidence. Pragmatic trial designs attempt to approximate real‐world conditions, while guideline developers frequently attempt to incorporate stakeholder, particularly patient stakeholder, perspectives when crafting their recommendations.^15^ The EtD framework proposed by the GRADE coalition attempts to go further. It offers a nuanced approach that anticipates the complexity of specific needs, circumstances and potential obstacles to implementation within different population subgroups and settings.^18^ However, the idealized contextualization achieved through such efforts remains upstream from practical decision‐making; it does not obviate the importance of, nor can it substitute for, the many processes that govern real‐world practice. For clinicians to consider adopting recommendations that differ from their current practice, they must reconcile them with the pre‐existing nexus of knowledge, experience, social relations and personal and institutional practice that already guide them. They incorporate such new knowledge by transforming it as they integrate it into their mindlines. That is, it is not just practitioner behaviour but the guideline recommendations themselves that are transformed in the course of being adapted to the living context.^9^ Hence, guideline recommendations are inevitably and necessarily provisional, that is, conditional.

## 4 SUGGESTIONS FOR GUIDELINE DEVELOPERS

The term ‘conditional’ is deliberately borrowed from the GRADE lexicon. There it is used to denote recommendations that a guideline panel considered to be weak due to inadequate research evidence, concerns about equity, resource availability and/or stakeholder values. To summarize our argument, the implementation of all recommendations depends upon the social and relational processes that necessarily govern decision‐making for individual patients. We propose, therefore, that even strong recommendations by guideline panels should ultimately be understood as conditional in the wider sense.

Even without new global health emergencies, the rate of change in clinical practice is increasing. Recent innovations on the part of researchers and guideline methodologists that attempt to match that acceleration include trial emulation using large databases drawn from medical‐record data,^27^ the creation of living guidelines and systematic reviews,^28^ and accelerated approaches to guideline development.^29^ Such advances may help decrease the discordant timeframes of research and practice. However, they cannot obviate the contextualization gap we have described. To address the latter, we offer some suggestions:

- Distinguish between highly researched, stable questions for which practice is unlikely soon to change and less researched, rapidly evolving topics that relate to ever‐changing practice and local context. The former may be suitable for the relatively rare strong recommendation; but the latter may ultimately be more useful to practitioners, despite being only provisional.
- Reassess the trade‐off between a highly robust research‐evidence base and a pragmatic, timely reliance on the best available forms of clinical evidence. Encourage rigorous and reliable, but rapid, methods for assessing a wide range of evidence.
- In tackling health disparity and equity, recognize that, although it may be possible to provide variations in guidance for some widespread variations in disease manifestations, many divergences and disparities are likely to be specific to local context and practice and therefore unresponsive to global recommendations.
- Take account of how published guideline recommendations are *actually* being interpreted and transformed when used within different healthcare contexts and populations. Explicit adherence to a guideline recommendation may not be obvious in a practice audit even though the guidance may significantly influence clinical practice. A nuanced interpretation of guideline uptake needs to supplant reliance on conventional compliance data.

In summary, adjustments are needed in the ways that clinical guidelines are developed, labelled and monitored, and in the time required for that effort, if they are to maximize their impact on the care of patients through access to ongoing research. This process has never been more important than it is today.

## AUTHOR CONTRIBUTIONS

Peter C. Wyer developed the governing concept of the manuscript, oversaw all aspects of development of the draft and its revisions and made final editorial decisions regarding the final submission. John Gabbay contributed original content to the manuscript and helped formulate, review, edit and revise the entire submission. Edward H. Suh contributed to all aspects of the design and flow of the manuscript and helped review, edit and revise the entire submission.

## CONFLICT OF INTEREST STATEMENT

The authors declare no conflict of interest.

## ETHICS STATEMENT

No ethical approval was needed for this study.

## Data Availability

Data sharing is not applicable to this article as no new data were created or analysed in this study.
